# Inflammation-Mediated Memory Dysfunction and Effects of a Ketogenic Diet in a Murine Model of Multiple Sclerosis

**DOI:** 10.1371/journal.pone.0035476

**Published:** 2012-05-02

**Authors:** Do Young Kim, Junwei Hao, Ruolan Liu, Gregory Turner, Fu-Dong Shi, Jong M. Rho

**Affiliations:** 1 Barrow Neurological Institute, Medical Center, St. Joseph’s Hospital, Phoenix, Arizona, United States of America; 2 Department of Neurology, Tianjin Medical University General Hospital, Tianjin, China; 3 Keller Center for Imaging Innovation, Barrow Neurological Institute, Phoenix, Arizona, United States of America; 4 Departments of Pediatrics and Clinical Neurosciences, University of Calgary, Alberta Children’s Hospital, Calgary, Alberta, Canada; Institute Biomedical Research August Pi Sunyer (IDIBAPS) - Hospital Clinic of Barcelona, Spain

## Abstract

A prominent clinical symptom in multiple sclerosis (MS), a progressive disorder of the central nervous system (CNS) due to heightened neuro-inflammation, is learning and memory dysfunction. Here, we investigated the effects of a ketogenic diet (KD) on memory impairment and CNS-inflammation in a murine model of experimental autoimmune encephalomyelitis (EAE), using electrophysiological, behavioral, biochemical and *in vivo* imaging approaches. Behavioral spatial learning deficits were associated with motor disability in EAE mice, and were observed concurrently with brain inflammation. The KD improved motor disability in the EAE model, as well as CA1 hippocampal synaptic plasticity (long-term potentiation) and spatial learning and memory (assessed with the Morris Water Maze). Moreover, hippocampal atrophy and periventricular lesions in EAE mice were reversed in KD-treated EAE mice. Finally, we found that the increased expression of inflammatory cytokines and chemokines, as well as the production of reactive oxygen species (ROS), in our EAE model were both suppressed by the KD. Collectively, our findings indicate that brain inflammation in EAE mice is associated with impaired spatial learning and memory function, and that KD treatment can exert protective effects, likely via attenuation of the robust immune response and increased oxidative stress seen in these animals.

## Introduction

Clinical symptoms in multiple sclerosis (MS), an autoimmune inflammatory disorder of the central nervous system (CNS), not only include motor disability but also cognitive deficits. Cognitive impairments are primarily learning and memory difficulties in 43% to 70% of MS patients during both early and late stages of MS [1]. Recent brain magnetic resonance imaging (MRI) studies in patients with MS have shown structural derangements of both the cerebral cortex and the hippocampus (a primary locus of memory consolidation). Moreover, convincing evidence of memory dysfunction in MS patients has been reported [2]–[4].

Experimental autoimmune encephalomyelitis (EAE), a commonly employed animal model of MS – characterized by inflammation and neurodegeneration in the CNS – has been shown to recapitulate hippocampal injury and synaptic loss with resultant impairment in spatial learning and memory [5]. Indeed, the extent of memory decline, more than the degree of motor disability, has proven to be a significant predictor of social handicap in MS [6]. One of the hallmark features of MS/EAE is immune-mediated CNS inflammation [7], [8], which subsequently facilitates reactive oxygen species (ROS) generation in a feed-back manner [9], [10].

Intriguingly, it has been observed that learning and memory decline in models of Alzheimer’s disease and normal rodents is accompanied by a cytokine burst [11]–[13], whereas cytokine blockade prevents such cognitive impairment [11], [13]. Further, it is known that ROS generation that exceeds cellular antioxidant capacity leads to disruption of hippocampal synaptic plasticity [14]. Despite the growing literature supporting the critical pathogenic role of inflammatory cytokines and chemokines, and aberrant ROS production in EAE/MS, it is unknown whether these factors are directly involved in contribution to memory loss in both humans and in the EAE model.

Dietary treatments have rapidly emerged as an alternative intervention for a variety of neurological disorders. Specially, the ketogenic diet (KD) has been shown to be clinically efficacious and safe in patients with medically refractory epilepsy [15]. The broad neuroprotective properties of the KD have been highlighted in a growing number of experimental models. For example, the KD attenuated amyloid beta 40 and 42 deposition in a mouse model of Alzheimer’s disease [16] and restored motor deficits in transgenic amyotrophic lateral sclerosis (ALS) mice [17]. Additionally, either inflammation or thermal nociception was significantly attenuated by KD treatment [18]. While the mechanisms underlying such effects remain unclear, one possibility is that the KD decreases ROS production by increasing the expression and activity of mitochondrial uncoupling proteins [19].

In the present study, we asked whether inflammatory responses and oxidative stress in a murine model of EAE are linked to memory dysfunction in mice. We found that impairment of spatial learning and memory *in vivo* and synaptic plasticity *in vitro* correlated closely with increased cytokine/chemokine expression and ROS generation. Further, we observed that the KD significantly dampened motor disability, CNS inflammation and memory dysfunction in EAE mice, suggesting that dietary interventions may prove to be clinically useful in the treatment of MS.

## Methods

### EAE Induction

C57BL/6 (B6) mice purchased from Taconic Farms were housed in an animal facility. The mice were 6–8 wk of age at the beginning of the study, and both standard and KD were given ad lib. EAE was induced by subcutaneous injection of myelin oligodendrocyte glycoprotein (MOG)_35–55_ peptide emulsified with complete Freund’s adjuvant (CFA) containing 500 µg of non-viable *Mycobacterium tuberculosis*, followed by intravenous injection of 20 ng of pertussis toxin in C57BL/6 (B6) mice. After EAE induction, the change in motor function was monitored daily using an arbitrary scale: 0, no symptoms; 1, flaccid tail; 2, hindlimb weakness or abnormal gait; 3, complete hindlimb paralysis; 4, complete hindlimb paralysis with forelimb weakness or paralysis; 5, moribund or deceased. The KD (Bio-Serv F3666 diet, Frenchtown, NJ, USA; 6.3∶1 ratio of fats: carbohydrate+protein) was fed to a separate cohort of mice 7 days before EAE induction and was continued until the time of sacrifice. All animal handling protocols were approved by the Institutional Animal Care and Use Committee (IACUC) at the Barrow Neurological Institute and St. Joseph’s Hospital & Medical Center (Protocol Number 0277).

### Magnetic Resonance Imaging (MRI)

Cerebral imaging was performed on a 7 T rodent MRI scanner using a 20 mm RF-Quadrature-Volume head coil. Axial and coronal T1-weighted (MSME; TE 10.5 ms, TR 322 ms, 0.5 mm slice thickness, matrix 256×256, field of view [FOV] 2.8 cm, eight averages, 40 coronal slices, scan time 22 minutes, and 20 axial slices, scan time 16 min), fat-suppressed turbo spin echo T2-weighted (RARE; TE1 14.5 ms, TE2 65.5 ms, TR 4500 ms, 0.5 mm slice thickness, Matrix 256×256, FOV 2.8 cm, eight averages, 40 coronal slices, scan time 28 minutes, and 20 axial slices, scan time 28 minutes) MRI data were analyzed using the MEDx3.4.3 software package (Medical Numerics, VA, USA) on a LINUX workstation.

### Morris Water Maze Testing

Spatial learning and memory were assessed using the standard Morris water maze (MWM), consisting of a circular tank (diameter, 56 in) filled with opaque water by the addition of non-toxic white paint (Fresco Powder tempera, Northvale, NJ) and a hidden platform in a fixed spot. Visual cues were placed in each of the four arbitrarily defined quadrants of the tank. During 5 days of initial training, the mice were dropped in the water maze in one of the four equal quadrants labeled north, east, south and west. Their order of use was changed daily. The mice were given four 90-second training trials per day. Regardless of success or failure to reach the hidden platform, all mice were given an additional 30-sec on the platform and then placed in its heated cage until its next trial. After initial training, the mice were placed in the tank and allowed to swim for 90 seconds to search for the submerged platform (4 daily trials over 5 consecutive days). The path lengths and latencies to platform discovery were measured using the EthoVision XT video tracking system (Noldus Information Technology, Leesburg, VA, USA).

### Electrophysiology

Based on changes in motor disability after EAE induction, the alteration of long-term potentiation was tested at two time-points (e.g., peak and mild disability). To address this, transverse hippocampal slices (400 µm) were prepared from brains of three groups of mice [control mice (naïve), standard diet (SD)-fed EAE and ketogenic diet (KD)-fed EAE] using a standard vibratome (The Vibratome Company, St. Louis, MO, USA) at either post-natal days 14–19 or 31–40. Slices were stored in oxygenated physiological saline (composition in mM: 124 NaCl, 1.8 MgSO_4_, 4 KCl, 1.25 NaH_2_PO_4_, 26 NaHCO_3_, 2.4 CaCl_2_, and 10 D-glucose; pH = 7.4) prior to recording. Each slice was then transferred to a recording chamber attached to an Axioskop FS2 microscope (Carl Zeiss Microimaging, Inc., Thornwood, NY, USA) and superfused with warm (34±1°C) physiological saline at a rate of 2–3 ml/min before the start of each experiment. Upon Schaffer collateral (SC) stimulation, excitatory postsynaptic potentials (EPSPs) were recorded at a control test frequency of 0.05 Hz (0.1 ms, 20–100 µA) from CA1 stratum radiatum. After establishing a standard input-output curve (i.e., stimulus intensity *vs.* EPSP amplitude), the baseline EPSP amplitude (over 1 mV) was set to 50% of maximum responses. High-frequency stimulation (HFS, 1 sec, 100 Hz) or theta-burst stimuli (TBS, consisting of 5 trains delivered at 0.2 Hz) were used to induce long-term potentiation (LTP, an electrophysiological measure of memory consolidation). LTP was expressed as the percent of the mean baseline EPSP amplitude during 5–10 min without HFS. We also used TBS-induced LTP to confirm the changes in synaptic plasticity seen with HFS. Recorded data were filtered at 3 kHz, sampled at 10 kHz using pClamp, and analyzed with Clampfit (Axon Instruments, Union City, CA, USA). Chemicals used in electrophysiological study were purchased from Sigma-Aldrich (St. Louis, MO, USA).

### CNS Cell Isolation and Flow Cytometry

To purify CNS cells, mice were transcardially perfused with phosphate buffer saline (PBS). Mononuclear T cells were collected from the brains of 5 mice using Percoll density gradients (30-70%). Single cell suspensions (4×10^5^ cells) were prepared and stained for cell surface markers of the following antigens (targeted by the indicated Antibody (Ab) fluorescently tagged with FITC, PE, allophycocyanin, PE-Cy5, or PE-Cy7): CD3, CD4, CD8, CD11b, CD19 and CD45. All Abs were purchased from BD Pharmingen. FACS analysis was performed on a FACSAria™ system using Diva software (BD Biosciences, CA, USA).

### Measurement of Cytokines and Chemokines

Both brain and spinal cord tissues were homogenized with PBS mixed with Halt protease inhibitor. After sonication, samples were centrifuged at 4°C for 20 min at 13,000 rpm. Isolated supernatants were mixed with a cocktail of reagents from a multi-cytokine and chemokine assay kit (Multi-Analyte ELISArray Kit; SA biosciences, USA) following the manufacturer’s instructions. Optical density was measured at 450 nm wavelength using a microplate reader (BioRad Model 680, Hercules, CA, USA).

### *In vivo* ROS Measurement

To evaluate the magnitude of CNS ROS changes *in vivo* after EAE induction with and without KD treatment, dihydroethidium (DHE, 10 mg/ml, Molecular Probes, Eugene, OR, USA) was dissolved in DMSO and injected intraperitoneally at a dose of 27 mg/kg. Mice were anesthetized with an initial dose of 5% isoflurane, followed by a maintenance dose of 2.5% isoflurane, during bioluminescent imaging of the live mice which was conducted over a 1 min acquisition times using an *in vivo* imaging system (Xenogen IVIS Spectrum, Caliper Life Sciences, Hopkinton, MA, USA). Signal intensities from the brain were defined and measured in the efficiency mode with the Xenogen system.

### Statistics

Numerical data were expressed as the mean ± SEM. The Mann-Whitney *U* test, student’s t-test and one-way ANOVA followed by Tukey test were used to assess differences and variance amongst different experimental groups. Significance was set at *p<*0.05.

## Results

### The Ketogenic Diet Ameliorates Motor Disability and Cognitive Impairment in EAE Mice

**Figure 1:**
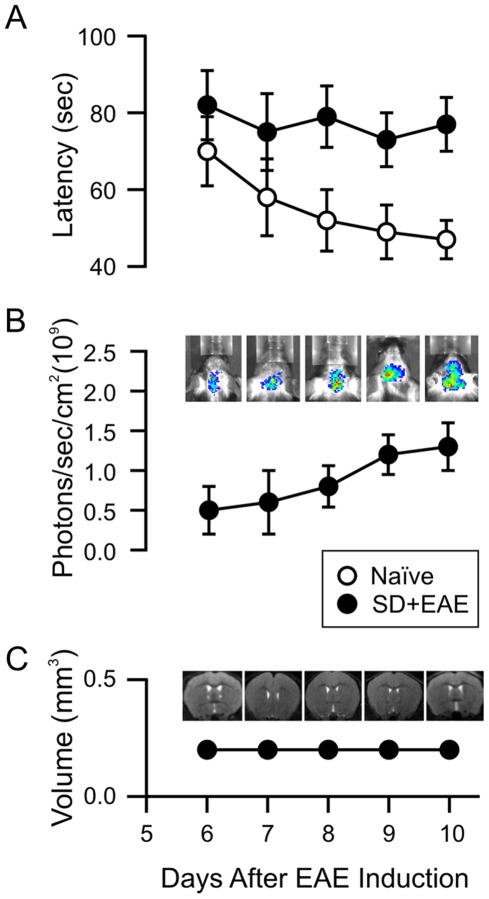
Changes in EAE mice prior to motor disability. (A) Assessment of spatial learning and memory using the Morris Water Maze (MWM) test. Naïve mice exhibited improved latencies to solve the maze during the consecutive 5-day trials, but EAE mice did not show any improvement in spatial learning and memory. (B) *In vivo* measurement of reactive oxygen species (ROS) after immunization. No change in ROS signal was found in naïve mice, but the progressive increases in superoxide production were seen 6–10 days post immunization. (C) No brain structural changes are seen in the early stage of immunization. All experiments were conducted in groups of mice (n = 4–8) between 6–10 days post immunization before gross motor deficits were observed.

**Figure 2:**
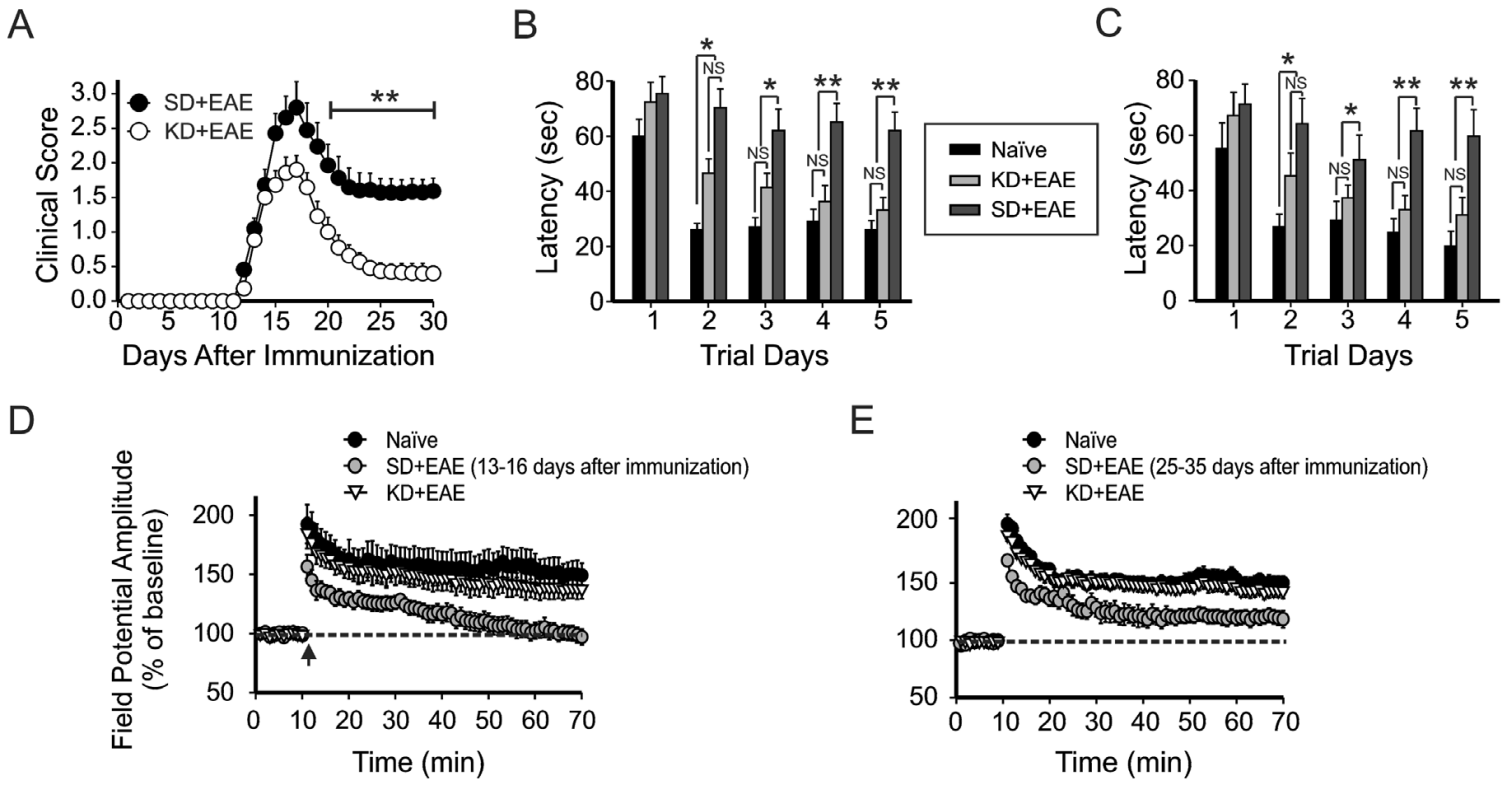
Effects of the ketogenic diet on motor disability and memory deficit in EAE mice. (A) Starting from day 17 post immunization (p.i.), KD-fed EAE mice (n = 11) showed significantly less motor disability than SD-fed EAE mice (**, *p<*0.01, Mann-Whitney *U* test). In KD- fed and SD- fed EAE mice, the mean clinical scores (1.9±0.20 vs 2.7±0.35 and 0.43±0.12 vs 1.0±0.15) were measured at 17 days and 25 days p.i., respectively. (B, C) Restoration of spatial learning and memory by the KD in EAE mice. MWM testing was conducted in groups of EAE mice either prior to the occurrence of motor deficits (before 10 days p.i., left panel, B) or from 30 days to 35 days p.i. (right panel, C). The graph illustrates average latencies to finding the submerged escape platform. Each group consisted of 5–8 mice. Values represent mean±SEM. One way ANOVA followed by Tukey test. (D) Change in the synaptic plasticity at 13–16 days p.i. with or without the KD. EAE mice exerted impairment in LTP after high-frequency stimulation (10 slices form 5 mice). Both the naïve and KD-fed EAE mice demonstrated intact LTP induction and maintenance (9 slices from 4 mice). (E) Change in synaptic plasticity at 25–35 days p.i. with or without the KD. Neither the naïve nor KD-fed EAE mice showed changes in LTP maintenance (10 slices from 5 mice). The dotted line indicates the baseline field potential amplitude. Synaptic plasticity seen during peak and mild stages of motor disability are depicted in the left and right panels, respectively.

Consistent with a previous clinical report indicating that sub-clinical memory impairment is not associated with motor disability in benign MS patients [20], we found a lack of motor dysfunction and visible brain MRI changes after immunization but prior to evidence of EAE onset. However, the progressive decline in spatial learning and memory function correlated inversely with ROS levels in the brain of EAE mice (**Figure 1**). EAE mice exhibited monophasic neurological symptoms of acute disseminated encephalomyelitis 10 days following immunization (**Figure 2A**). Using our previously reported motor disability scale, where higher numbers reflect greater impairment [21], the mean clinical score was 2.8±0.37 at 17 days post EAE induction and remained stable thereafter (at about 1.2) 20 days post EAE. In KD-fed EAE mice, disability at both the peak stage (14–19 days) and mild recovery stage (25–35 days) was significantly decreased compared to SD-fed EAE mice (*p*<0.01); the clinical score measured 1.9±0.2 and 0.39±0.1 at 17 days and 30 days post EAE, respectively (**Figure 2A**).

During the initial immunization period before EAE onset, KD treatment prevented a decline in behavioral spatial learning and memory (**Figure 2B**). No significant differences were found between the latencies of SD-fed naïve mice and KD-fed EAE mice in finding the hidden platform during the MWM test. The protective effects of the KD in this paradigm were sustained at 25–35 days post EAE induction (**Figure 2C**). Additionally, motor activity as monitored by swim speed was affected by changes in motor disability in EAE mice; naïve mice swam significantly faster than SD-fed EAE and KD-fed EAE mice on day 5 of the trial. In naïve, KD-fed EAE and SD-fed EAE mice, the speed scores (23±3.2, 34±5.8 and 37±6.8 cm/sec, respectively) were measured at 17 days after EAE induction. Intriguingly, recovery of speed was seen when EAE mice were kept on the KD; no difference between naïve and KD-fed EAE mice was found at 25 days of EAE induction (20±4.5 *vs* 24±5.6 cm/sec, respectively). Consistent with this, there was a significant increase in the latencies of SD-fed EAE mice to finding the hidden platform as compared to the SD-fed naïve mice or KD-fed EAE mice (*p*<0.01).

Based on these findings, we examined the changes in synaptic plasticity in EAE mice with SD or KD treatment by measuring CA1 hippocampal LTP at two different time-points (14–19 days *vs.* 30–35 days). Measuring LTP is the optimal way to evaluate memory function in the EAE model due to motor disability and the possibility of optic neuritis which would preclude accurate behavioral assessments [21], [22]. SD-fed naïve mice exhibited intact LTP induction and maintenance at both stages (**Figure 2 D, E**); the EPSP amplitude measured 147±9% and 146±5% at 60 min post-HFS or TBS, respectively. In SD-fed EAE mice at 60 min post-HFS or TBS, despite the slightly increased EPSP amplitude at this stage of mild motor disability, the EPSP amplitudes were significantly different from those seen in both SD-fed naïve and KD-fed EAE mice (**Figure 2** D, E). In contrast, LTP was fully restored in KD-fed EAE mice at both stages. Values were not significantly different compared to those obtained from SD-fed naïve mice (*p*<0.05).

### The Ketogenic Diet Reverses Structural Brain Lesions in EAE Mice

**Figure 3:**
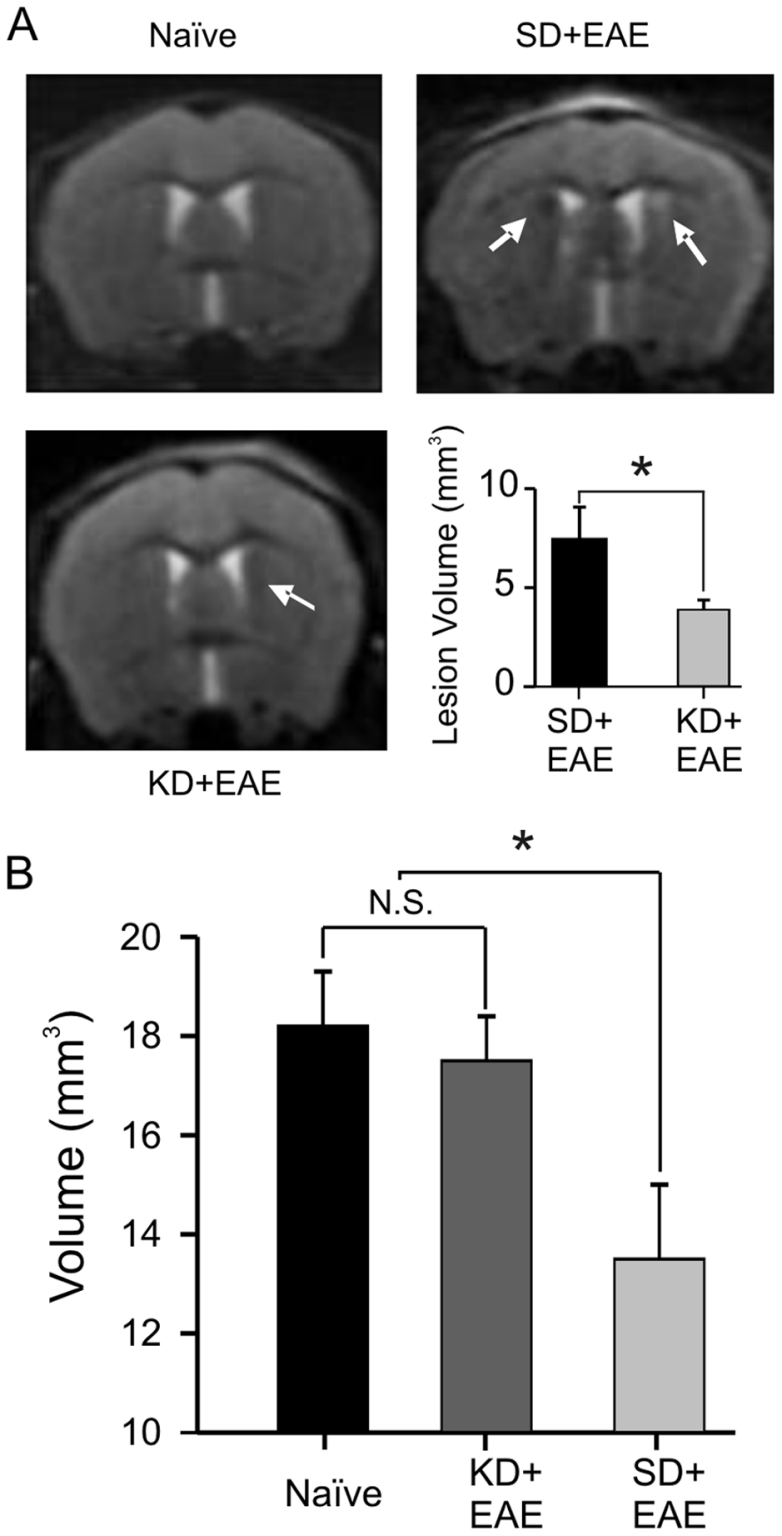
Structural brain changes in EAE mice with and without KD treatment. (A) T2-weighted images were obtained with a 7T MR scanner during the peak stage of motor disability. EAE mice showed abnormal signal intensities adjacent to the lateral ventricles. Arrows indicate focal lesions on T2-weighted imaging. In contrast, lesion volumes were significantly reduced in KD-fed EAE mice (N = 4–8). (B) In contrast to the observed hippocampal volume loss in EAE mice at 30 days post immunization, neither naïve mice nor KD-fed EAE mice showed changes in hippocampal volume.

To investigate whether the functional impairments noted above are associated with changes in lesion size in brains of EAE mice, we employed high-field animal MR imaging to assess EAE mice fed either the SD or the KD. Notably, our findings were similar to clinical data demonstrating periventricular lesions and hippocampal atrophy seen in patients with MS-induced cognitive/memory decline [3], [23]. We found that the periventricular region exhibited areas of abnormal signal intensity at the peak stage of motor disability post EAE immunization as compared to naïve mice (**Figure 3A**). In contrast, KD-fed EAE mice showed significantly reduced lesion signal intensity compared to SD-fed EAE mice. While we were not able to find any hippocampal structural changes at 15 days post immunization, T2-weighted imaging detected hippocampal volume loss at 30 days post immunization. Importantly, KD-fed EAE mice did not show any hippocampal volume loss, consistent with our behavioral and electrophysiological data, and no significant differences in hippocampal volume were found between naïve mice and KD-fed EAE mice (**Figure 3B**).

### The Ketogenic Diet Diminishes CNS Inflammation and Oxidative Stress in EAE Mice

**Figure 4:**
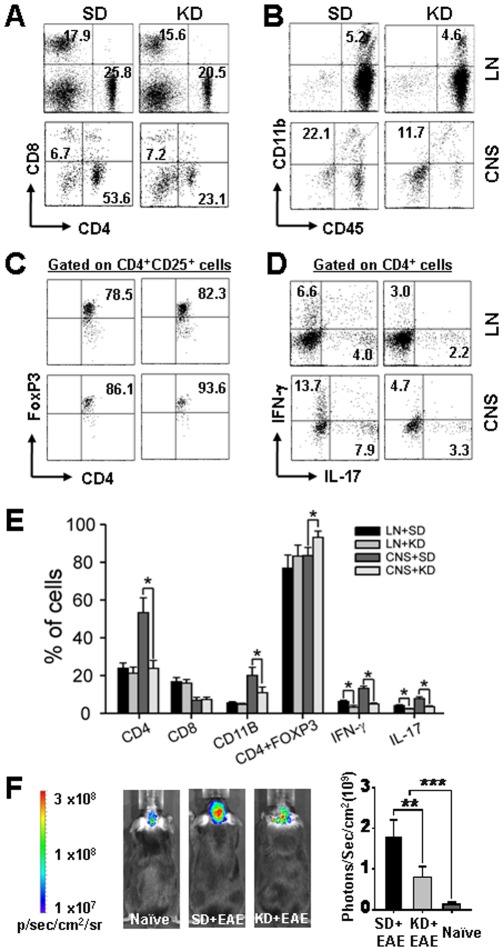
The KD ameliorates EAE-mediated CNS-inflammation and oxidative stress. Comparison of the frequencies of (A) CD4^+^/CD8^+^ T cells, (B) CD11b^+^/CD45^+^ cells on gated lymphocyte populations in EAE with and without KD treatment. (C) Frequencies of CD4^+^CD25^+^ Foxp3^+^ regulatory T cells. The KD suppressed CNS cellular infiltration and myelin-reactive T cell responses in EAE. Groups of mice (receiving standard diet [SD] and KD diet) were immunized with MOG/CFA/PT, and sacrificed between day 16–19 p.i. Lymph node, spleen and CNS cells were isolated. (D) Lymphoid (LN) or CNS cells were re-stimulated with MOG_35–55_ peptide overnight and IFN-γ- and IL-17-expressing CD4^+^ T cells were measured by intracellular staining. The number seen in the box indicated the average percentage of individual markers on the cells. (E) Summary data of the percentage of individual marker in the cells (LN *vs.* CNS) in SD-treated EAE mice or KD-treated EAE mice. Data are representative of two independent experiments (n = 4–8/group). P values, Student’s *t*-test; *, *p*<0.05. (E) Visualization and quantification of brain inflammation by *in vivo* bioluminescence imaging. This is achieved by imaging reactive oxygen species (ROS) levels in the brain. Bioluminescent images in live mice were captured during a 1 min acquisition time using the Xenogen IVIS system at several time points after injection of 27 mg/kg dihydroethidium. Representative images of ROS seen in the left panel were captured in naïve mice and EAE mice with and without KD treatment. All experiments were conducted on groups of mice (n = 4–8) between days 14–19 p.i. Data are representative of two independent experiments (mean and SEM). P values, one way ANOVA; **, *p*<0.01; ***, *p*<0.001.

Given both functional and structural protective effects of the KD in our EAE model, we next asked if these beneficial changes were a consequence of modulating T cell-mediated inflammatory responses. KD-fed mice had a 2–2.5 fold reduction in CNS-derived CD4^+^ cells and CD11b^+^CD45^+^ cells (macrophage and microglia), compared to SD-fed mice (**Figure 4A, B**). Consistent with the potential therapeutic role of regulatory T (T_reg_) cells against various autoimmune diseases [24], KD treatment in EAE mice resulted in a tendency toward increased CD4^+^CD25^+^Foxp3^+^ T_reg_ cells (**Figure 4C**). As shown in Fig 4D, KD-fed mice also exhibited down-regulation of CD4^+^ cells with a statistically significant matched reduction in the expression levels of cytokines (IL-1β, IL-6, TNF-α, IL-12, IL-17) and chemokines (IFN-γ, MCP-1, MIP-1α, MIP-1β), compared with SD-fed EAE mice in the periphery and in the brain (**Table 1**).

**Table 1:** Inflammatory mediators assayed in EAE mice.

Cytokine/chemokine		*IL-1 β*	*IL-6*	*TNF-α*	*IL-12*	*IL-17A*	*IFN-γ*	*TGF-β*	*MCP-1*	*MIP-1α*	*MIP-1β*
***CNS***	***EAE***	33.3±5.8	211±36.5	50.2±11.3	18.2±3.8	131±22.3	75.6±11.9	116±26.0	42.6±6.1	666±80.8	200.6±46.3
	***EAE+KD***	11.5±3.8*	50.6±8.7**	25.2±5.1*	12.2±2.1*	40.1±4.3**	23.9±3.3*	90.4±19.1	15.7±2.6*	226±50.8**	110.1±21.3*
***Periphery***	***EAE***	50.3±9.3	99.2±11.8	37.6±5.8	11.8±1.9	152±15.4	27.1±6.0	179±20.9	56.3±8.9	1061±95.9	353±36.4
	***EAE+KD***	22.5±4.7*	57.6±4.7*	20.7±3.7*	6.24±0.7*	70.9±11.0*	20.1±5.6	175±11.1	33.9±7.5*	789±106.3*	216±30.0*

Lymph node and CNS homogenates were prepared from mice in the indicated groups at day 15 p.i. Cytokine concentrations were measured by a Multi-Analyte ELISArray Kit (SA Biosciences, MD). Unit: pg/ml; n = 4/group. P values, student’s *t*-test or Wilcoxon test;

** , *p*<0.01;

* , *p*<0.05.

To clarify the functional relationship between ROS generation and memory dysfunction, we directly measured changes in *in vivo* brain ROS levels after intraperitoneal DHE injection. Consistent with our initial finding of increased ROS production in the early period following EAE induction (**Figure 1**), a significant increase in superoxide radical levels were seen in the brains of SD-fed EAE mice at the peak stage of motor disability, as compared to levels observed in SD-fed naïve mice and KD-fed EAE mice. As expected, KD treatment strongly suppressed ROS levels in EAE brains, but the ROS intensity was significantly different from that seen SD-fed naïve mice (*p*<0.01) (**Figure 4E**).

## Discussion

The principal findings of the present study are: (1) both brain inflammation and oxidative stress are associated with disruption of memory consolidation in the EAE model of MS; (2) memory dysfunction in EAE appears independent of structural brain changes prior to the onset of motor disability (as detected with high-field MR imaging); (3) at the onset of EAE, impairment of synaptic plasticity and spatial learning/memory are coincident with both motor disability and the appearance of brain lesions; (4) the KD exerts strong suppressive effects on EAE-induced motor and memory dysfunction, but does not prevent initial EAE onset; and (5) the neuroprotective effects of the KD may be mediated by a reduction in lymphocyte proliferation, cytokine/chemokine expression and oxidative stress.

There is mounting evidence that an aberrant cytokine and chemokine burst characterized by T-cell proliferation may be a critical factor in the initiation of MS/EAE [7], [8]. Although memory impairment has been shown to result from increased cytokine expression (e.g., IL-1β, IL-6 and TNF-α) in patients with Alzheimer’s disease, vascular dementia and post-operatively [25], [26], the functional role of inflammatory mediators in MS/EAE-mediated memory impairment has yet to be fully elucidated. Our findings strongly suggest that CNS inflammation plays a major role in the memory impairment seen in EAE. Furthermore, the broad modulation of inflammatory cytokines and chemokines by the KD appears linked to the diet-induced attenuation of adverse functional and structural consequences in EAE. We found that KD treatment increased the differentiation of T_reg_ cells and led to a concomitant decrease in IL-6 and T_h_17 cell generation in EAE, changes that are consistent with a recent study reporting similar findings in MS, and proposed to yield beneficial effects [27]. Collectively, our data strongly support the notion that the KD may be clinically useful in patients with MS.

It is well known that up-regulated cytokine/chemokine expression triggers reactive oxygen species (ROS) production, which subsequently promotes an inflammatory cascade in a feedback manner [10], [28]. The link between neuroinflammation and pathologic ROS production is further supported by a prior study demonstrating elevations in thiobarbituric acid substances and F2-isoprostane levels (both indicators of oxidative stress) in the cerebrospinal fluid of MS patients [29]. Additionally, ROS generated by microglia and macrophage activation has been correlated with demyelination and axonal injury in both EAE and MS [10], [30], [31].

Consistent with these studies, our data clearly indicates that *in vivo* ROS generation is associated with impaired memory consolidation post EAE induction. The KD not only significantly reduces CNS-derived CD4^+^ cells and CD11b^+^CD45^+^ cells, but also restores memory function in EAE mice possibly by reducing *in vivo* brain ROS levels. While this protective effect may be a consequence of decreased microglia and macrophage activation, we cannot exclude the possibility that the KD enhances intrinsic antioxidant capacity. Earlier studies have shown elevations in hippocampal glutathione peroxidase activity and catalase levels induced by the KD and its associated metabolites such as ketone bodies [32], [33].

MR imaging studies have helped solidify the concept that the memory impairment seen in MS patients is closely related to structural brain changes [3], [4]. Notably, the CA1 hippocampal region in humans becomes atrophic in the early stages of MS which then spreads to adjacent areas [34]. This clinical observation is paralleled by a recent study demonstrating loss of neurons, synaptic integrity and memory impairment in EAE mice [5]. In the present study, hippocampal volume loss in EAE mice was also documented on MR imaging at the same time when impairment of memory consolidation was observed.

Many metabolic substrates [e.g., polyunsaturated fatty acids (PUFAs) and ketones] and hormones [e.g., leptin and adiponectin] [35]–[37] are both elaborated and altered by KD treatment. Interestingly, dietary supplementation with omega-3 PUFAs – which mirrors one important facet of the KD – led to decreased pro-inflammatory and enhanced anti-inflammatory changes in models of MS [38]. However, whether PUFAs are beneficial in MS patients remains controversial [39]–[42].

There are other metabolic players that may be relevant to EAE. It is well known that both adiponectin increases and leptin decreases after chronic calorie restriction. Thus, it is of interest that calorie restriction – which shares some metabolic features with the KD – exerted anti-inflammatory and neuroprotective effects in EAE [43]. Prior studies have demonstrated that up-regulation of adiponectin suppressed inflammatory mediators (e.g., TNF-α, IL-1, IL-6, and IL-10) and led to weight loss [44], whereas increased leptin expression correlated with a pro-inflammatory action [45]. Additionally, KD-treated obese rats showed increases in adiponectin expression, weight-loss and down-regulation of TNF-α levels [37]. Thus, it appears that a metabolic treatment such as the KD or calorie restriction induces changes in specific hormones that positively modulate neuroinflammation.

Another potential pathogenic mechanism in MS is hypoxia-induced mitochondrial dysfunction in excitable demyelinated axons, which leads to decreased ATP production [46]. Furthermore, down-regulation of mitochondrial uncoupling protein (UCP) activity in EAE –which results in pro-inflammation and ROS generation – was shown to induce serious motor disability [47]. In this light, it is interesting to note that the KD has been shown to provide synaptic protective effects in hippocampal slices under conditions of low glucose-induced metabolic stress, and was correlated with up-regulation of genes encoding mitochondrial ATP synthases [48]. Similarly, a more recent study demonstrated that ketone bodies alone afforded synaptic protection against the effects of mitochondrial respiratory chain inhibitors via enhanced ATP generation and antioxidant activity [33]. Further, it has been shown that the KD increases the expression and activity of UCPs in normal brain – effects that led to diminished mitochondrial ROS generation [19]. Taken together, the experimental evidence would strongly suggest that bioenergetic stabilization and subsequent reduction in oxidative stress may yield neuroprotective effects in MS/EAE.

In summary, we have shown that memory deficits in EAE mice are correlated with brain inflammation as evidenced by the enhanced expression of inflammatory cytokines and chemokines. And importantly, the KD significantly attenuated brain inflammation and reversed both memory dysfunction and motor impairment in EAE mice. There is mounting evidence that metabolic substrates (and certain hormones) can reduce inflammatory responses, and as a consequence provide both structural and functional neuroprotective effects. Since the KD is currently available in many clinical centers throughout the world, patients with MS may readily benefit from this non-pharmacological treatment option. In light of the present study and supportive evidence from the literature noted above, prospective, controlled clinical trials assessing the therapeutic effects of the KD now appear warranted.
